# Advancements in Mixed-Matrix Membranes for Various Separation Applications: State of the Art and Future Prospects

**DOI:** 10.3390/membranes14110224

**Published:** 2024-10-25

**Authors:** Bhoga Arundhathi, Manideep Pabba, Shrisha S. Raj, Nivedita Sahu, Sundergopal Sridhar

**Affiliations:** 1 Membrane Separations Lab, Chemical Engineering and Process Technology Division, CSIR-Indian Institute of Chemical Technology, Tarnaka, Hyderabad 500007, TS, India; barundhati.knr@gmail.com (B.A.); pmanideep682@gmail.com (M.P.); shrishas1995@gmail.com (S.S.R.); nivedita@csiriict.in (N.S.); 2 Academy of Scientific and Innovative Research (AcSIR), Ghaziabad 201002, UP, India

**Keywords:** MMM, nanomaterials, chemical grafting, water treatment, gas separation, pervaporation

## Abstract

Integrating nanomaterials into membranes has revolutionized selective transport processes, offering enhanced properties and functionalities. Mixed-matrix membranes (MMMs) are nanocomposite membranes (NCMs) that incorporate inorganic nanoparticles (NPs) into organic polymeric matrices, augmenting mechanical strength, thermal stability, separation performance, and antifouling characteristics. Various synthesis methods, like phase inversion, layer-by-layer assembly, electrospinning, and surface modification, enable the production of tailored MMMs. A trade-off exists between selectivity and flux in pristine polymer membranes or plain inorganic ceramic/zeolite membranes. In contrast, in MMMs, NPs exert a profound influence on membrane performance, enhancing both permeability and selectivity simultaneously, besides exhibiting profound antibacterial efficacy. Membranes reported in this work find application in diverse separation processes, notably in niche membrane-based applications, by addressing challenges such as membrane fouling and degradation, low flux, and selectivity, besides poor rejection properties. This review comprehensively surveys recent advances in nanoparticle-integrated polymeric membranes across various fields of water purification, heavy metal removal, dye degradation, gaseous separation, pervaporation (PV), fuel cells (FC), and desalination. Efforts have been made to underscore the role of nanomaterials in advancing environmental remediation efforts and addressing drinking water quality concerns through interesting case studies reported in the literature.

## 1. Introduction

**Figure 1:**
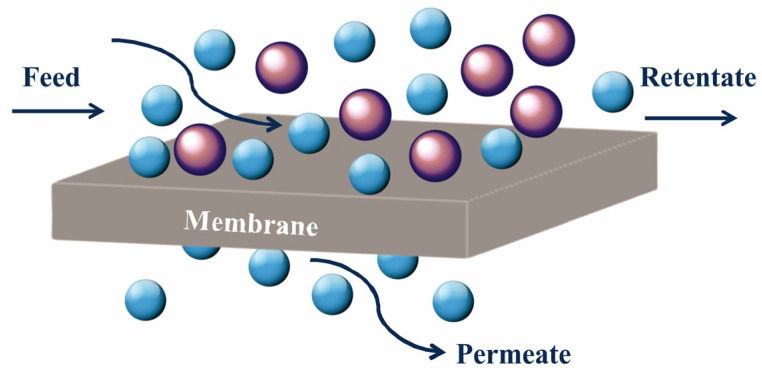
General concept of the membrane filtration process.

A membrane essentially functions as a barrier, separating two phases while selectively controlling the transport of different components. Their classification spans diverse criteria, including membrane material, morphology, geometry, and preparation techniques. Synthetic membranes exhibit a broad spectrum, ranging from organic (polymeric) to inorganic (ceramic/metal), with variations in solidness, electrical charge, and structural symmetry. Their geometric shapes vary from flat to tubular or hollow fiber membranes (HFMs), each tailored for specific applications. Membranes serve various functions, from separating mixture compositions to preventing permeation. Moreover, membrane processes are categorized based on the driving force applied, encompassing pressure-driven methods like reverse osmosis (RO), nanofiltration (NF), and gas separation, along with concentration gradient-driven processes like dialysis and temperature-driven techniques such as membrane distillation (MD). Additionally, electrical-potential-driven processes like electrodialysis offer further versatility in membrane applications [1]. The schematic of the general membrane separation method is shown in Figure 1; however, the working mechanism for each membrane application may differ. In mixed-matrix membranes, several modes of mass transfer are possible, such as solution diffusion within the dense polymer phase, while in the fillers, molecular sieving, Knudsen’s diffusion, viscous flow, capillary condensation, and pore adsorption phenomena may also occur [1].

In 2021, the global membrane separation technology market reached a significant value of USD 22.08 bn, showcasing a remarkable growth trajectory anticipated to continue at a projected rate of 12.2% from 2022 to 2030. This growth surge is primarily fueled by the escalating stringency of environmental regulations governing wastewater management practices and the escalating demand for seawater desalination solutions worldwide. Moreover, industries such as dairy processing, food, and beverage increasingly embrace membrane separation technologies to address purification challenges and meet stringent quality standards. Notably, ongoing advancements in membrane durability and antifouling properties further bolster these technologies’ adoption across various sectors, promising enhanced efficiency and sustainability in diverse applications [2].

**Figure 2:**
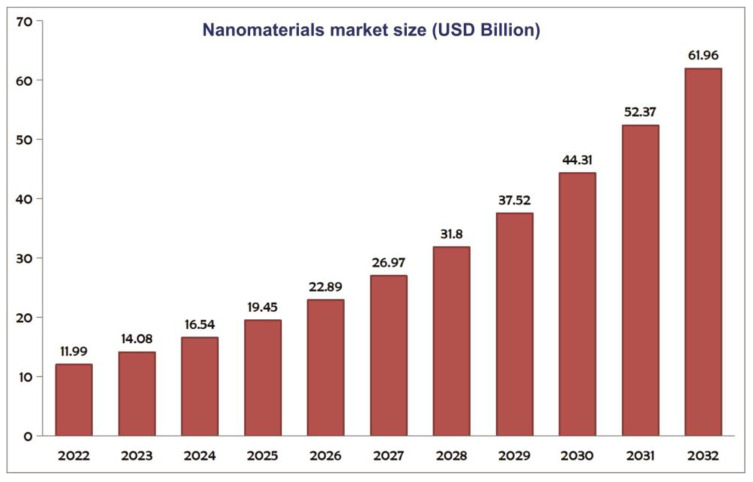
Market growth of nanomaterials [3].

Likewise, the global nanomaterials market, valued at $11.99 billion in 2022, is poised to soar to $61.96 bn by 2032, demonstrating a strong compound annual growth rate of 17.90%. This surge is primarily catalyzed by the remarkable physiochemical properties of nanoparticles (NPs), fueling their extensive utilization across diverse sectors, including healthcare, aerospace, and textiles. Despite facing hurdles in 2020 amid the COVID-19 pandemic, the market witnessed a resilient resurgence in 2021. This rebound was propelled by a combination of government-imposed restrictions and financial support measures, coupled with the introduction of pioneering applications in various technologies, collectively driving the nanomaterials sector’s unprecedented growth trajectory [3]. Figure 2 depicts the anticipated market expansion of nanomaterials in the coming years, highlighting the projected growth trajectory of this dynamic sector.

The dynamic growth of the nanomaterials sector commonly intertwines with membrane technology, where they share a symbiotic relationship as both barriers and facilitators of selective transport processes.

Membranes can be crafted from various organic (polymers) and inorganic (ceramics) substances. However, polymeric materials garner extensive attention due to their superior physical strength, chemical steadiness, and adaptability. Polysulfone (PSF) is the most prevalent membrane material, prized for its exceptional chemical and thermal stability [4]. Researchers are also exploring and utilizing a range of polymers like polyvinylidene fluoride (PVDF), polyacrylonitrile (PAN), polyethersulfone (PES), polyvinyl chloride (PVC), polyvinyl alcohol (PVA), polyethylene (PE), polyamide (PA), and polypropylene (PP), among others [5].

Over the past few decades, membrane technology has garnered rising attention due to its numerous advantages, including a smaller footprint, cost-effectiveness, durability, eco-friendliness, reduced chemical usage, ease of scalability, and minimal formation of chemical sludge, among others [6]. However, despite its many advantages, membrane technology has some limitations. For example, fouling and sensitivity to chlorine can significantly reduce the lifespan of membranes, particularly in pressure-driven separation processes [7,8]. This phenomenon results in a turndown in permeation flux, alterations in selectivity, and reduced separation capability for a period of filtration operations, ultimately diminishing the overall lifespan of the membrane [5]. Researchers have investigated embedding various nanomaterial additives in polymers to enhance hydrophilicity, reduce membrane fouling, and improve performance. This approach offers greater control over membrane structure and fouling reduction. Nanomaterials such as MWCNTs, GO, ZnO, copper (Cu), silver nanoparticles (Ag), Al_2_O_3_, ZrO_2_, SiO_2_, and TiO_2_ have garnered significant interest in wastewater treatment applications [9]. Concentration polarization represents a significant drawback observed in non-porous membranes during concentration-driven or electrical-driven processes. This phenomenon results in the addition of solutes close to the membrane, leading to reduced efficiency and potentially affecting the process’s overall performance [10]. Various NPs offer promising solutions, including metals, metal oxides, carbon nanomaterials, zeolites, and MOFs. When membranes are made up of these NPs or nanomaterials, they are termed nanocomposite membranes (NCMs). Integrating NPs into the polymeric matrix enhances membrane properties and performance, offering a versatile approach to overcoming existing challenges in membrane technology [11]. Incorporating NPs into the polymeric matrix significantly modifies the membrane’s structural, morphological, mechanical, thermal, antimicrobial, and antifouling properties. This integration enhances the versatility and performance of the membranes, offering a multifaceted approach to address various challenges encountered in membrane applications [12,13,14]. 

Mixed-matrix membranes (MMMs) containing various nanoparticles (NPs) exhibit notable antifouling properties through several mechanisms. Carbon nanotubes (CNTs) provide exceptional biofouling resistance due to their intrinsic cytotoxic properties that inhibit microbial growth, making them ideal for desalination applications. TiO_2_ is favored for its stability in harsh conditions, ease of fabrication, and commercial availability. Including NPs in the dope solution enhances hydrophilicity, reduces fouling, and improves composite membranes’ thermal and mechanical stabilities.

ZnO nanoparticles embedded in polymeric membranes form a stable system that retains physical properties and chemical activity, offering a novel antifouling solution. Alumina nanoparticles (Al_2_O_3_) are used in membrane modification for filtration due to their stability, hydrophilicity, and robust mechanical properties. Graphene oxide (GO) is a promising nanomaterial for developing antifouling nanocomposite membranes due to its hydrophilic nature, low toxicity, antibacterial properties, and cost-effectiveness. GO is a suitable filler for desalination, water purification, gas separation, and pervaporation. It exhibits strong electrostatic repulsion toward organic solutes like bovine serum albumin (BSA), preventing adsorption. Additionally, GO nanocomposite membranes demonstrate enhanced self-cleaning and antibacterial properties against microbes, including antibacterial activity against *Salmonella typhi*, a gram-negative bacterium that causes typhoid [9,15].

NCMs find applications in various separation processes, including gas–gas, liquid–liquid, and liquid–solid separations. They have emerged as a promising solution for desalination, addressing the traditional tradeoff between permeability and solute rejection while minimizing fouling. This technology represents a significant advancement in developing high-performance membranes for desalination, marking a crucial step toward the next generation of membrane manufacturing [16].

### 1.1. Fundamentals of Mixed-Matrix Membranes (MMM)

**Figure 3:**
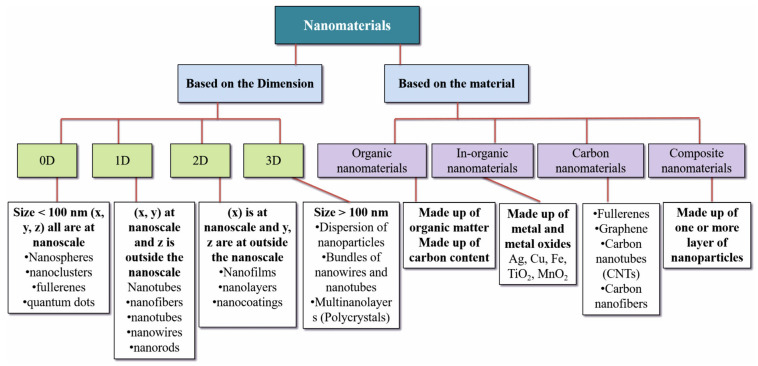
Classification of NPs.

MMMs are advanced composite membranes of an organic polymer matrix embedded with inorganic or organic fillers. The primary aim of integrating these fillers is to merge the favorable properties of both components, thereby enhancing the whole membrane’s performance, particularly in selectivity and permeability. The polymer matrix in MMMs serves as the continuous phase, providing structural integrity and flexibility. Common polymers include polyimides, PSFs, and cellulose acetate, known for their excellent film-forming properties and stability. The dispersed fillers, on the other hand, can be inorganic materials like zeolites, metal organic frameworks (MOFs), silica, and carbon nanotubes, or organic fillers like porous aromatic frameworks (PAFs) and covalent organic frameworks (COFs) [17]. Figure 3 presents a concise categorization of NPs. The fillers contribute to the membrane’s functionality through several mechanisms. They can act as molecular sieves, selectively allowing specific molecules to pass through based on size and shape. This selective permeability is crucial for applications like gas separation and water purification. Additionally, fillers can disrupt the polymer matrix, creating microcavities that enhance gas and liquid permeability. However, the relationship between the polymer matrix and the nanofillers is critical; poor compatibility may lead to non-selective voids and reduced membrane performance [18].

Recent advancements focus on improving the compatibility between the polymer and the fillers through surface modifications and structural adjustments. This includes functionalizing the filler surfaces to improve their complexion with the polymer matrix, thereby reducing defects and improving overall separation performance. Innovations in this field have shown promise in extending the lifespan of MMMs and achieving higher separation efficiencies, making them viable for industrial applications [17]. MMMs are advanced materials used for various separation processes, combining polymers with inorganic or organic fillers to enhance performance. The types of fillers and polymers commonly used in MMMs are as follows.

### 1.2. Types of Fillers

Zeolites: Microporous aluminosilicate minerals known for their huge surface area and chemical strength, commonly used for gas separation and catalysis.

Metal–organic frameworks (MOFs): These consists of metal ions or clusters coordinated with organic ligands, MOFs feature high porosity, structural tunability, and low density, making them excellent for gas storage and separation.

Zeolite imidazolate frameworks (ZIFs): A subclass of MOFs with imidazole linkers, offering changeable pore sizes and superior thermal, chemical, and moisture steadiness compared to other MOFs.

Oxide NPs: These includes non-porous silica, metal oxide NPs, mesoporous silica, and polyhedral oligomeric silsesquioxanes (POSS), used for their stability and surface functionality.

Nanocarbons: These include carbon nanotubes (CNTs), graphene, carbon molecular sieves (CMS), and activated carbon, valued for their unique structural properties and high surface areas.

### 1.3. Types of Polymers

Glassy polymers: These are rigid polymers that provide high selectivity and are used for applications requiring sharp molecular sieving. Examples include but are not limited to polyimides (e.g., 6FDA-Durene), polyethersulfone (PES), polycarbonate (PC), polysulfone (PSF), polyamide (PA), and acrylonitrile-butadiene-styrene (ABS).

Rubbery polymers: Flexible polymers that offer high permeability and are used for applications needing high flux. Examples include but are not limited to polydimethylsiloxane (PDMS), polyether block amide (PEBA), polyvinyl acetate (PVAc), styrene-butadiene rubber (SBR), poly(vinylidene fluoride) (PVDF), and polyurethane (PU).

Integrating these fillers into polymer matrices results in MMMs that combine the mechanical strength and processability of polymers with the enhanced separation properties of inorganic fillers [19,20]. 

## 2. Recent Advances in MMM Fabrication Techniques

MMMs represent a cutting-edge development in membrane technology, combining polymer matrices with inorganic or organic fillers to enhance separation performance. These membranes leverage the complementary properties of polymers, such as flexibility and processability, with the superior mechanical, thermal, and chemical characteristics of fillers like NPs, zeolites, or MOFs. Integrating these materials can considerably improve the selectivity, permeability, and durability of the membranes, making them highly effective for gas separation, water treatment, and PV applications.

The fabrication of MMMs is a complex process that requires meticulous control to achieve uniform distribution of NPs and tough interfacial bonding with the polymer matrix. Various fabrication technologies have been formulated to optimize these parameters and tailor the membrane attributes for specific applications. This review provides an in-depth synopsis of the primary fabrication techniques for MMMs, including phase inversion, electrospinning, and layer-by-layer (LbL) assembly. The structural and functional characteristics of the resultant membranes are influenced by the particular benefits and difficulties associated with each technique. Understanding these techniques is crucial for advancing the design and application of high-performance MMMs in diverse industrial and environmental contexts.

### 2.1. Phase Inversion (PI) Physical Blending Technique

**Figure 4:**
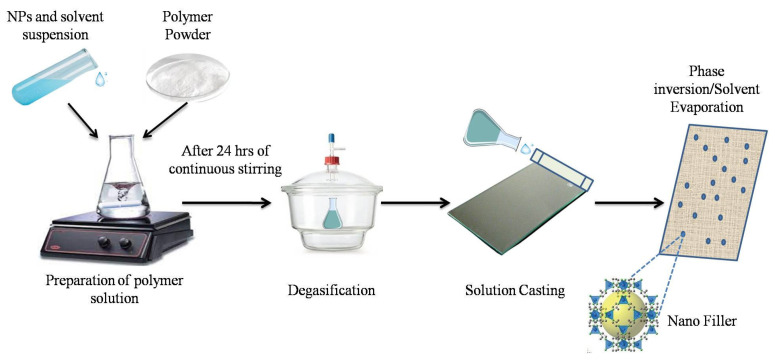
MMM fabrication by blending technique.

The phase inversion (PI) technique, also recognized as the Loeb-Sourirajan method, involves the controlled transfer of a polymer from the liquid phase to the solid phase [21]. This technique encompasses various approaches such as thermally induced phase separation (TIPS), evaporation-induced phase separation (EIPS), vapor-induced phase separation (VIPS), and immersion precipitation. The immersed precipitation method is the widely employed phase inversion technique, which includes two main types: hollow-fiber and flat-sheet [22]. Xiaoyu Tan et al. used innovative techniques like modified EPIS to develop high-performance MMMs. These membranes outperformed both polymeric and zeolite-only membranes, showcasing robustness and scalability for cost-effective use in various gas and liquid separations, especially with challenging zeolites [23]. NPs-loaded MMMs can be effectively prepared using the PI technique, a widely used technique in membrane fabrication. This method involves creating a uniform solution of polymer along with NPs, which is then cast into a thin film. Then the layer is subsequently placed in a non-solvent coagulation bath, inducing phase separation and forming a porous membrane structure. A schematic representation of the blending technique utilized to prepare MMM is shown in Figure 4. The incorporation of NPs such as titanium dioxide (TiO_2_), montmorillonite (MMT), or other metal oxides enhances the membrane’s properties, including mechanical strength, hydrophilicity, and antifouling capabilities [24,25].

For instance, embedding MMT nanoclays into cellulose acetate membranes through phase inversion has significantly improved the separation efficiency and antifouling properties. The resultant MMMs exhibit superior performance in oil–water separation with enhanced water flux and rejection rates, demonstrating the practical advantages of nanoparticle incorporation via the PI technique [24]. This method’s versatility and efficacy make it crucial to advancing membrane technology for various filtration applications.

#### 2.1.1. Phase Inversion Method for Fabricating Flat Sheet NCMs

Flat sheet NCMs are commonly produced using the phase inversion method, allowing precise control over membrane thickness to suit specific applications. The fabrication process involves manually pouring a nanoparticle-loaded polymer solution onto a support with an automated casting machine. This is followed by dipping in an opposite solvent (typically water or a water/solvent mixture) to induce phase separation and create a porous membrane structure. After phase inversion, the substrate is carefully removed from the glass plate and soaked in DI water for further use [26]. For dense membranes, the solvent evaporation technique is used. In this method, the casting solution is dried at ambient temperature under atmospheric conditions until a dense membrane forms [27]. 

Various researchers have demonstrated the versatility of the PI method. For example, Moghadam et al. incorporated TiO_2_-NPs into a solution of 20 wt% polyvinylidene fluoride (PVDF) and five wt% polyethylene glycol (PEG) in dimethylacetamide (DMAc) solvent using non-solvent induced phase separation (NIPS) and 20 min of sonication at 20 °C [28]. Efome et al. created flat-sheet NCMs with SiO_2_-NPs by varying the nanoparticle weight percentage and using a membrane thickness of 0.25 mm for VMD [29]. Maheswari et al. prepared NF membranes by incorporating silver NPs (Ag-NPs) into polyether ether sulfone (PEES) dissolved in *N*-methyl-2-pyrrolidone (NMP) solvent [30]. These membranes, set at 18% relative humidity and 25 ± 2 °C, were allowed to gel for about 30 s before immersion in a non-solvent bath containing 2 wt% NMP, 4g sodium lauryl sulfate (SLS), and 2L DI water.

These examples illustrate the adaptability of the phase inversion method in fabricating flat sheet NCMs with tailored properties for various filtration and separation applications.

#### 2.1.2. Fabrication of Hollow Fiber Membranes (HFMs) Using Nanoparticle Incorporated Dope Solutions

The transformation of NP-loaded dope solutions into HFMs is achieved through a spinneret and a hollow fiber spinning machine. HFMs are typically prepared using four main methods: (1) melt spinning, (2) dry spinning, (3) wet spinning, and (4) a combination of dry and wet spinning. In these techniques, polymers or polymer blends are passed through a spinneret nozzle, forming the core with water or other liquids such as ethanol [31]. A critical aspect of these methods is maintaining an air gap between the non-bath and the fibers.

Commercially, these capillary fibers have inner and outer diameters exceeding 25 µm and less than 1 mm, respectively, making them suitable for applications in medicine, wastewater treatment, gas separation, and photovoltaic processes. HFMs offer significant advantages over flat sheet and spiral wound membranes, including higher area per unit volume, higher recovery rates, and the capability of self-supporting individual units [32]. Various studies have demonstrated the benefits of these methods. Hebbar et al. engineered functionalized Fe_2_O_3_-NP incorporating HFMs using the dry-wet spinning method via the phase inversion technique to enhance bio-fouling resistance [33]. Saberi et al. developed SiO_2_-NP-incorporated polyvinyl chloride (PVC) HFMs through the dry-jet wet spinning method, employing 15 wt% dimethylformamide (DMF) as the bore liquid and adjusting polymer and NP percentages [34]. Turken et al. synthesized HFMs by incorporating Ag-NPs into PES polymer using the dry-wet phase inversion technique, using DI water as the bore liquid, maintaining a 15 cm air gap, and spinning the fibers at a collecting speed of 4.82 m/min [35]. These examples highlight the versatility and effectiveness of various spinning methods in producing HFMs with tailored properties for diverse industrial applications.

### 2.2. Electrospinning

**Figure 5:**
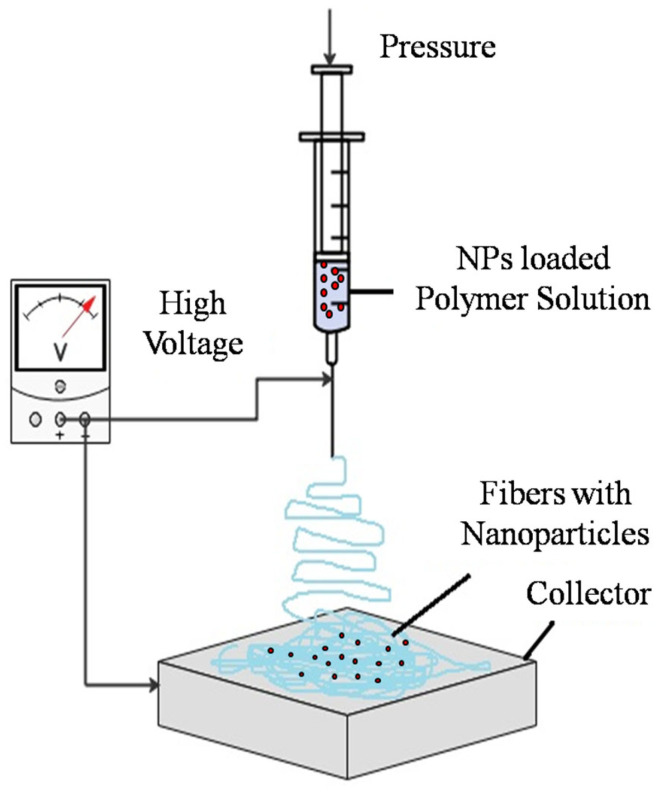
Electrospinning method for MMM fabrication.

The electrospinning technique is a highly efficient and versatile method for producing nanofibers, widely adopted across various fields such as water filtration, tissue engineering, drug delivery, battery technology, indoor air filtration, and facial masks. Its ease of modification, cost-effectiveness, and adaptability make it a preferred choice for fabricating small-diameter fibers. Electrospinning’s effectiveness is influenced by several parameters, including polymer characteristics, additives, tip-collector distance, feeding rate, and applied voltage. This method has been used to create a wide range of nanofibers for indoor air filters from polymers like PES, polyurethane (PU), polyacrylonitrile (PAN), polyvinyl chloride (PVC), polycarbonate (PC), polyvinyl alcohol (PVA), and polyamide 6 (PA-6) [36]. Electrospinning involves a high-voltage power supply, a syringe pump, a needle, and a conductive collector. A polymer solution is pumped through a syringe, forming charged droplets that create a jet directed toward the collector. Upon drying, these droplets solidify into fibers, as shown in Figure 5. This method’s advanced control over process parameters allows for tailoring properties, expanding its application in energy storage, membrane technology, drug delivery, and tissue engineering. Besides polymers, electrospinning can also be applied to metals and ceramics, chosen for their molecular weight, volatility, and solvent conductivity [37]. 

Recent studies have highlighted electrospinning’s versatility and scalability. For example, Elmarghany et al. created three-layered nanocomposite membranes through electrospinning, incorporating PES-carbon nanotubes and poly(vinylidene fluoride-co-hexafluoropropylene). This membrane exhibited enhanced porosity and hydrophobicity, making it suitable for MD applications and offering a cost-effective alternative with competitive performance [38]. 

Furthermore, Tawsif et al. developed NF membranes for arsenic removal using PSF composite materials with graphene oxide (GO) and zinc oxide (ZnO). The inclusion of GO and ZnO improved porosity, hydrophilicity, and surface negative charge, resulting in superior water permeability and doubled arsenite removal compared to pristine PSF membranes [39]. These advancements demonstrate the significant potential of electrospinning for fabricating high-performance nanofibrous membranes for diverse industrial applications.

### 2.3. Layer-by-Layer (LbL) Self-Assembly

LbL assembly is a commonly used method for coating substrates with various materials, offering precise control and versatility compared to other thin-film deposition techniques. Initially involving the sequential adsorption of oppositely charged materials, LbL assembly has expanded to include diverse molecular interactions, such as biotin-streptavidin binding. This technique has garnered significant research interest owing to its feasible applications in biomedicine, separations, and drug delivery [40].

Guo et al. developed high-performance composite NF membranes using tannic acid (TA) and Jeffamine (JA) via LbL assembly, inspired by mussel adhesion. These membranes, constructed without substrate pre-treatment, exhibited high pure water flux (37 L m^2^ h^−1^ bar^−1^) and over 90% dye rejection for molecular weights between 269 and 1017 g mol^−1^. The selective layer’s hydrophilic surface and covalent bonds ensured outstanding antifouling properties and long-standing performance [41]. 

Ahmad et al. utilized an accelerated LbL assembly technique via spraying to enhance RO TFC membranes. By alternating layers of titania nanosheets (TNS) and polyethyleneimine (PEI), they achieved the highest water permeability (1.39 m^2^ h^−1^ bar^−1^) and a 19.42% increase over dip-coated membranes, while maintaining a 97% salt rejection rate. Furthermore, the 1PEI/TNSSC-TFC membranes showed outstanding antifouling properties against BSA and NaAlg, achieving 100% permeate retrieval after physical cleansing [42]. The LbL self-assembly technique is used to construct multilayers of polyelectrolytes containing charged species such as nucleic acids, proteins, dyes, and viruses. This approach entails the step-by-step adsorption of oppositely charged species via electrostatic attraction, resulting in the formation of multilayer structures [43]. Harsini et al. highlighted the homogeneous scattering of nanomaterials inside the polymer matrix and controlled interfacial interactions, underscoring the method’s simplicity, versatility, and effectiveness [44]. Dhar et al. noted that LbL assembly can create ultrathin advanced surface coatings through forces like hydrogen bonding, electrostatic interactions, charge transfer, covalent bonding, and van der Waals interactions. The covalently bonded LbL self-assembly process, a relatively new area of research, offers additional stability and robustness, making it suitable for harsh conditions [45]. Liu et al. used this technique to incorporate calcium oxalate (CaOx) NPs into membranes for dehydration via PV, demonstrating its versatility in enhancing membrane performance [46]. Persistent challenges in membrane technology stem from the limited compatibility between inorganic and organic fillers and the polymer matrix, leading to reduced selectivity and non-selective voids at the interface. Recent advancements have introduced novel fillers like MOFs, PAFs, and porous organic cages (POCs), which offer customizable structures to enhance permeability and selectivity. Surface chemical modifications and structural adjustments further optimize interactions between fillers and matrices, improving separation performance. Despite MOF’s favorable compatibility, their structural instability necessitates the exploration of porous organic fillers with regular channels, exceptional interface compatibility, and high chemical stability to prolong membrane lifespan and enhance separation efficiency [47]. Li et al. proposed a novel fabrication method for dye removal using MOFs. They deposited MOFs onto porous substrates via filtration, forming dense layers, and sealing interstices through in-situ polymerization of monomers. Using the zeolitic imidazolate framework (ZIF)-8 with hydrophobic butyl methacrylate and gently hydrophilic glycidyl methacrylate, they achieved 42% ZIF-8 loading. The developed membranes showed CR dye rejection rates of 97% for ZIF-8/BMA and 60% for ZIF-8/GMA, underscoring the superior performance of the hydrophobic and dense poly-BMA sealant [48].

Zhao et al. developed porous amino-functionalized nanosheets (PEI-F-Ce) and PEI-F-Ce/polyethylene oxide (PEO) MMMs using electrostatic self-assembly. They adjusted the lateral and pore sizes of the nanosheets by varying the PEI concentration. MMMs containing 2% PEI-F-Ce-2.5 nanosheets exhibited outstanding CO_2_ permeability (641 Barrer) and CO_2_/N_2_ selectivity (70.1), outperforming pure PEO membranes by 62% and 53%, respectively. These stable pore channels enhanced CO_2_ transport and adsorption capacity, significantly improving CO_2_/N_2_ selectivity, especially at cryogenic temperatures [49]. Incorporating KAUST-7, a fluorinated MOF with selective 1D channels, into a polyimide matrix produces molecular sieving MMMs. Surface functionalization of KAUST-7 NPs improved interfacial compatibility, reducing non-selective defects. With up to 45 wt% MOF loading, the resulting membrane achieved a propylene permeability of approximately 95 Barrer and a propylene/propane selectivity of around 20, maintaining structural stability under harsh conditions, highlighting the significance of surface engineering for advanced MMMs in industrial applications [50]. Overall, LbL assembly is a powerful tool for fabricating advanced membranes, offering precise control over layer composition and thickness, adaptability to various materials and interactions, and expanding potential applications across multiple industries.

## 3. Performance Enhancement Strategies

Performance enhancement in MMMs involves innovative strategies to overcome inherent material limitations and improve separation efficiency. By integrating advanced fillers like MOFs, PAFs, and POCs, and employing techniques such as surface functionalization and structural adjustments, researchers aim to optimize the interaction between fillers and polymer matrices. These advancements not only boost permeability and selectivity but also enhance the stability and lifespan of MMMs, making them more effective for a range of industrial applications.

### 3.1. Matrix-Level Modification of Membranes

**Figure 6:**
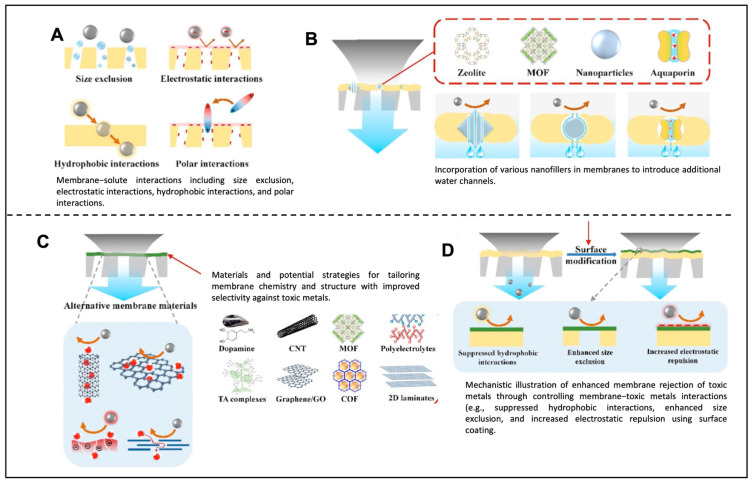
(**A**) Diagram illustrating the interactions between membranes and solutes. (**B**) The addition of nanofillers improves both water permeability and the removal of toxic metals. (**C**) Modification of membranes for increased selectivity by altering their structure and chemistry. (**D**) Mechanisms of toxic metal removal by membranes. Adopted from [52] with permission.

**Figure 7:**
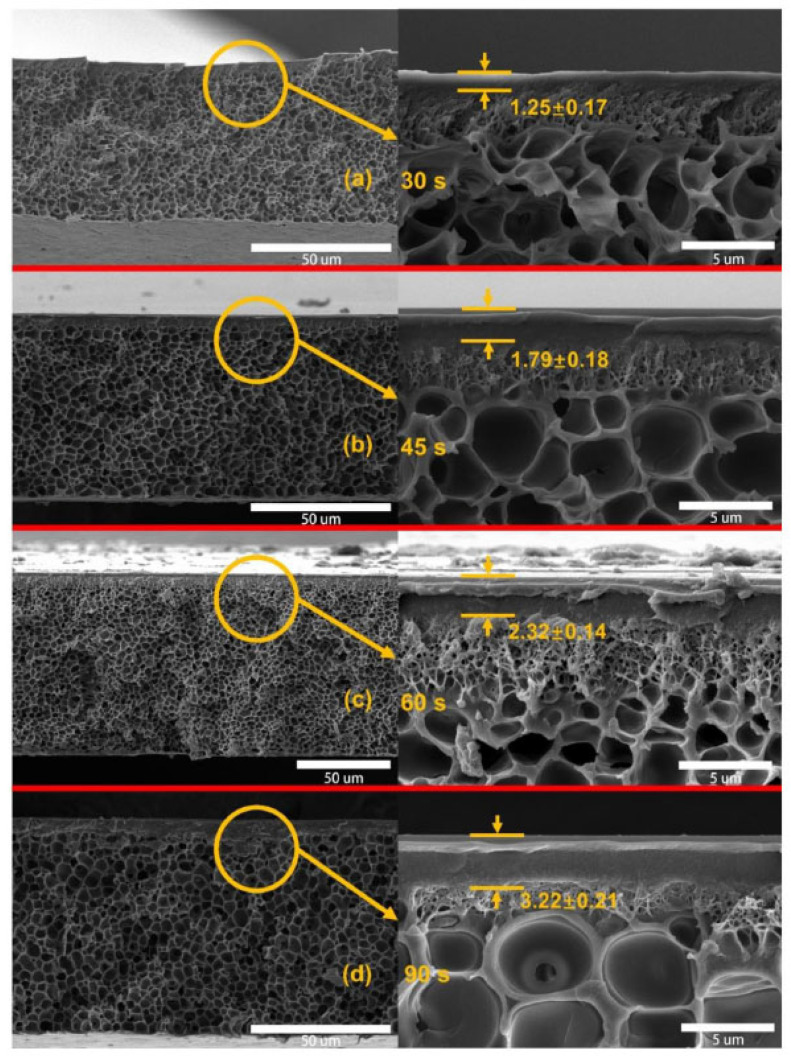
Scanning electron microscopy (SEM) images showing the cross-sections of asymmetric polysulfone (PSF) membranes at various evaporation times: (**a**) 30 s, (**b**) 45 s, (**c**) 60 s, and (**d**) 90 s. (Reproduced from Ref. [55] with permission).

In membrane-based separations, there is often a trade-off: increased permeation of a chemical can lead to decreased membrane selectivity. To overcome this, scientists employ various strategies, like two-dimensional materials, cross-linked polymers, zeolites, metal NPs, coated nanofibers, carbon nanotubes, or other nanomaterials. The pore size distribution in membranes determines which chemicals can be divided. Solute transport through a polyamide NF/RO membrane depends on solute partitioning into the rejection layer, influenced by size exclusion, electrostatic interactions, and various physicochemical interactions with the polymeric matrix. Incorporating different nanofillers into TFN membranes introduces additional water channels, such as intrinsic nanochannels within porous nanofillers, induced nanochannels around hydrophilic nanofillers, and selective water channels mimicking aquaporins, enhancing water transport efficiency [51]. Figure 6 depicts interactions between membranes and solutes, highlighting nanofiller incorporation for improved water permeability and toxic metal removal. It also illustrates potential modifications for enhancing membrane selectivity through structural and chemical alterations and mechanisms for rejecting toxic metals [52]. Nanomaterials offer tortuous paths for separation, as seen in recent applications like using multi-walled carbon nanotube meshes to filter particulate matter and metals from cigarette smoke. Similarly, loading zeolites onto nanoporous palladium membranes enhances selectivity. Fabricating low-tortuosity paths in ceramics improves flux and selectivity by controlling surface access [53]. The presence of fillers disrupts polymer chains, increasing free volume in glassy polymers. This phenomenon, often explained by tortuosity theory, creates convoluted paths for gas penetration. Consequently, smaller gas molecules like CO_2_ pass through MMMs faster than larger ones such as CH_4_ and N_2_. However, the literature suggests that high filler concentrations lead to filler particle aggregation within the polymer matrix during membrane preparation, adversely affecting membrane performance. This limitation restricts the scalability of MMMs for industrial gas separation applications [54]. Mei’s study revealed that coating MMMs (comprising ZIF-8 and PSF) with PDA repaired surface defects, leading to decreased H_2_ permeability but improved H_2_/CO_2_ selectivity. In the instance of PDA-2/10 wt% ZIF-8/PSF-30 MMM, the H_2_ permeability measured 23.3 Barrer, with an H_2_/CO_2_ selectivity of 9.3 at 30 °C under 4 bar pressure. Both H_2_ and CO_2_ permeabilities notably decreased as the solvent evaporation time increased, as evidenced by the enhanced thickness of the selective skin layer of PSF membranes in Figure 7 [55]. 

Dispersed particles impact gas permeability through several mechanisms. They can act as molecular sieves, selectively allowing gas molecules to pass based on their size. The introduction of particles can disrupt the polymeric matrix, creating additional microcavities and thereby enhancing permeability. Conversely, particles may also obstruct gas transport, reducing permeability. Effective membrane performance requires a strong affinity between the sieve particles and the polymeric phase [56]. 

### 3.2. Surface Modification of Membranes

Surface modification of membranes presents a promising method to impart novel properties to existing membranes. This process enhances separation properties, alters surface energies, and introduces chemical functionalities distinct from the bulk membrane materials. Modifications enable improved chemical resistance (e.g., fouling resistance and solvent resistance), controlled pore size, and defect elimination, leading to enhanced flux or selectivity [57]. Superhydrophilic polymeric membranes are typically created through surface coatings or grafting, but hydrophilic additives in coatings often lack adhesion to hydrophobic membranes, leading to washout. Surface grafting improves stability, and increasing the surface coverage ratio enhances antifouling properties. However, accessing the membrane surface with hydrophilic monomers is challenging, and their homopolymerization reduces coverage. Amphiphilic monomers offer a solution that is compatible with hydrophobic membranes. Recently, there has been significant interest in mussel-inspired surface chemistry for multifunctional coatings, with polydopamine (PDA) standing out due to its strong underwater adhesion and versatile reactivity. To simplify processes and enhance functionality, Zhang et al. introduced a one-step copolymerization using dopamine and unsaturated acrylate monomers [58]. This approach addressed challenges and enriched coating performance. Additionally, using reactive surfactants as unsaturated monomers offers both amphiphilic properties and high reactivity, allowing the successful construction of antifouling membrane surfaces in a single step by co-depositing dopamine and allyloxy nonylphenoxy propanol polyoxyethylene (SE-10N) ether ammonium sulfate (AHPS) under alkaline conditions [59]. Despite the potential benefits, MMMs face challenges hindering industrial application. Poor filler–polymer interfacial compatibility can cause voids and non-selective defects. Additionally, filler aggregation within polymeric matrices reduces separation performance. To overcome these challenges, efforts include surface modification of filler particles, priming protocols, thermal annealing, in situ MOF synthesis, and covalent grafting strategies [60]. 

The interaction between filler and matrix poses a significant challenge to composite functionality and application. This challenge has garnered attention for decades, with various approaches proposed to address it. Crucial to composite mechanical properties is a strong filler–matrix interaction. Researchers have recognized common challenges in composites and continue to investigate solutions. One challenge is the potential aggregation of hydrophilic fibers in hydrophobic matrices due to the numerous OH groups on their surface. The industrialization and broad use of fiber/matrix composites depend on achieving surface-chemical compatibility between hydrophilic cellulose fillers and hydrophobic matrices. Surface modification and compatibilization of fibers are effective ways to address this incompatibility. Chemical surface modification can enhance the hydrophobicity of nanocellulose, improving surface morphology, removing impurities, and promoting mechanical interlocking for better interfacial relationships and dispersion in hydrophobic media. Various surface treatment methods, such as heat, alkaline, plasma, and coupling treatments, have been used to reduce the surface polarity of fibers and fillers, enhancing interfacial interaction with hydrophobic polymer matrices [61]. Priming is a common method to enhance filler dispersion, where filler particles are thinly coated with polymer before mixing with the bulk polymer. This reduces stress at the filler/polymer interface, minimizing aggregation. However, priming alone may not eliminate particle agglomeration in MMMs with NPs. Another approach is interfacial polymerization, where inorganic fillers are dispersed with organic monomers, and polymerization occurs at the filler–monomer interface [62]. Surface modification of nanodiamonds with PEI was confirmed successfully through elemental analysis, XPS, and FTIR. Scanning electron microscopy (SEM) observations demonstrated enhanced interfacial adhesion and dispersion of nanodiamonds in the Pebax matrix with the presence of the PEI layer. Incorporating up to 1 wt% of ND-PEI filler in the MMMs notably improved the CO_2_/N_2_ selectivity, attributed to PEI’s “CO_2_ carrier” role [63]. 

## 4. Characterization Techniques for MMMs

Characterizing the structure, morphology, and performance of MMMs involves various analytical techniques. These techniques help understand the distribution and interaction of fillers within the polymer matrix, the membrane’s structural integrity, and its separation efficiency. Membrane characterization can be broadly categorized into three types: structural characterization, morphological characterization, and performance characterization. Here is an overview of some of the most commonly used analytical techniques:

### 4.1. Morphological Characterization

The morphological characterization of MMM involves analyzing the distribution and integration of fillers within the polymer matrix to ensure optimal performance. Techniques like scanning electron microscopy (SEM) and transmission electron microscopy (TEM) are commonly used. SEM offers intricate visuals of the membrane’s surface morphology and cross-sectional structure, while TEM offers high-resolution insights into the nanostructure and filler–polymer interfaces. These methods help understand fillers’ dispersion, compatibility, and potential agglomeration, which are critical for enhancing the membrane’s separation capabilities and mechanical properties.

#### 4.1.1. Scanning Electron Microscopy (SEM)

SEM is crucial for analyzing membrane morphology and topography, providing data on pore size and selective layer thickness. Unlike optical microscopes, SEM uses electrons instead of light, offering magnifications up to 1 million. Samples must be solid, electrically conductive, and often coated with gold or palladium to enhance image quality and prevent thermal damage. SEM excels in analyzing composite membranes and determining gas permeability in Barrer by measuring dense layer thickness. However, it is not suitable for pore sizes in gas separation membranes below tens of micrometers or thin films less than 10 nm thick. For such cases, higher resolution techniques like transmission electron microscopy are needed [64]. Additionally, field emission scanning electron microscopy (FESEM) offers even higher image resolution, particularly useful for examining particle–polymer interfaces in materials like glassy polymers [19]. Huang et al. demonstrated how important SEM is for assessing MMMs for gas separation. They investigated the dispersion of MOF filler in polymers of intrinsic microporosity (PIM-1) polymer solutions and found that ZIF-B@PIM-1 MMMs had better dispersion than their ZIF-8@PIM-1 counterparts, which improved membrane transparency and compatibility. Selective transport was hampered by filler aggregation and flaws in high-filler content ZIF-8@PIM-1 MMMs, in contrast to homogeneous MOF dispersion in low-filler content MMMs. In addition, the ZIF-8@PIM-1 MMMs with irregular polymer embossments demonstrated a moderate MOF-polymer connection. At the same time, the ZIF-B@PIM-1 MMMs showed well-dispersed fillers and strong filler–polymer bonding, supported by increased matrix affinity. SEM’s significant contribution to evaluating membrane morphology and efficacy is highlighted by its notable demonstration of little polymer deformation and robust filler–polymer adhesion in ZIF-B@PIM-1 MMMs [65]. In a study examined by Ting et al., membrane surfaces and elements in pristine, low-rGO-PVDF, and high-rGO-PVDF MMM were examined following 40 h of direct contact membrane distillation (DCMD) desalination. Initially, similar morphologies observed under FESEM changed with prolonged use, leading to salt deposition on all membranes. This salt, identified as Na and Cl through FESEM and energy-dispersive X-ray (EDX), showed varying weight percentages, with the highest in pristine, followed by low rGO-PVDF, and then high rGO-PVDF membranes. Rougher surfaces were associated with reduced salt deposition, enhancing antifouling properties. Moreover, while element O was detected in all membranes, its percentage increased in rGO-PVDF matrices, indicating rGO persistence post-desalination [66].

#### 4.1.2. Atomic Force Microscopy (AFM)

AFM has emerged as an effective technique for meticulously analyzing the surfaces of polymeric membranes with high resolution. AFM detects and records local interactions as it scans across the sample surface using a finely tapered pyramidal tip affixed to a cantilever. AFM offers two primary imaging modes: contact mode and tapping mode. The quantitative imaging (QI) mode also provides insights into membrane properties such as adhesion, elasticity, and surface topography. AFM enables the examination of various membrane attributes, including roughness, pore size, hydrophobicity, pore density, and distribution. Its adaptability to liquid and air environments makes it indispensable for researchers studying membranes in operational conditions [67]. In a study exploring the influence of carbon nanotube (CNT) diameter on MMMs, AFM analysis revealed that the standard roughness of PES, PES/CNT1, and PES/CNT2 membranes was 10.0, 10.8, and 10.5 nm, respectively. Despite a 0.1 wt% CNT concentration in both PES/CNT1 and PES/CNT2 MMMs, surface roughness had no significant difference. Wang et al. attributed the slight increase in roughness to CNT aggregation along with the higher viscosity of the polymer solution [68]. Moreover, Mukherjee et al. noted that the concentration of GO affects membrane surface roughness. GO 0.1, GO 0.2, and GO 0.5 membranes exhibited average roughness of 11, 17, and 24 μm, respectively, with higher GO concentrations resulting in greater height differences and increased roughness. AFM imaging confirmed GO accumulation on the membrane surface, facilitating phenomena such as adsorption and surface charge [69]. Furthermore, compared to membranes studied by Zhao et al., the developed membrane demonstrated lower surface roughness, indicating enhanced antifouling properties [70]. Additionally, Jainesh et al. observed a reduction in surface roughness upon incorporating GO-TiO_2_ fillers in PVC-MMMs. Similarly, studies by Rezaee et al. and Rasheed et al. found that composite membranes exhibited smoother surfaces compared to pristine membranes, as revealed by AFM characterization [9,71,72].

#### 4.1.3. Transmission Electron Microscopy (TEM)

TEM has been employed to scrutinize the complex structure and elemental composition of samples, boasting ultrahigh resolution compared to light microscopy. In the analysis of membrane-based materials, TEM provides nanoscale images of membranes and their constituent components. While obtaining cross-sectional and conducting imaging of membrane films is feasible, it requires meticulous preparation of thin samples. This method holds significant value in enhancing our comprehension of membrane fabrication mechanics, the relationship between material composition and performance, and strategies for mitigating membrane fouling [73]. 

### 4.2. Structural Characterization

Structural characterization of MMM involves utilizing various analytical techniques to understand the distribution, interaction, and compatibility of fillers within the polymer matrix. Techniques like X-ray diffraction (XRD) offer insights into the crystalline structure and phase distribution of the fillers, while FTIR and XPS help identify chemical interactions and elemental composition. Together, these methods ensure a comprehensive understanding of the MMM’s structural properties, which is crucial for optimizing performance in separation processes.

#### 4.2.1. X-Ray Diffraction (XRD)

XRD is a specialized method used to analyze the crystal structure of membranes, offering unique insights into the arrangement of polymeric chains and identifying whether the polymer is glassy or amorphous. Glassy polymers exhibit a well-ordered, crystalline structure, leading to enhanced mechanical properties, making them preferred due to their exceptional selectivity, particularly in gas separation applications, ensuring superior product purity. Conversely, amorphous polymers possess a disordered structure but offer high gas permeability because they lack diffusion limitations. In addition to crystal structure analysis, XRD can identify chemical compounds in samples, aiding in confirming sample purity and detecting secondary phases from membrane degradation. This technique utilizes a filament, elements such as tungsten are utilized to generate an X-ray beam directed towards the sample, and the scattered X-rays are analyzed to determine diffraction angles based on Bragg’s law, represented by Equation (1).

*nλ* = 2*dsinθ* (1)

Here, “*n*” typically represents the order of reflection, which is usually one. “*λ*” denotes the wavelength, determined by the radiation source, commonly copper K-α. “*d*” signifies the plane distance between a set of atoms.

XRD proves useful in analyzing the influence of introducing fillers into the polymer matrix. These fillers are expected to be identifiable within the XRD peak profile alongside the polymer [64].

#### 4.2.2. Fourier-Transform Infrared Spectroscopy (FTIR)

FTIR is a pivotal technique for characterizing MMM, as it provides detailed information on the chemical structure and interactions within the membrane. By analyzing the vibrational modes of molecular bonds, FTIR helps identify functional groups present in both the polymer matrix and the incorporated fillers. This technique is particularly useful for detecting any chemical interactions or bonding between the filler and polymer, which can significantly influence the membrane’s performance. For instance, shifts in characteristic absorption peaks can indicate the formation of new chemical bonds or changes in the local environment around specific functional groups. Additionally, FTIR can be used to monitor the distribution and compatibility of fillers within the polymer matrix, ensuring a uniform and well-integrated composite material. Such detailed chemical insights are crucial for optimizing the design and functionality of MMMs for various applications, including gas separation and water treatment.

FTIR is a vital characterization tool used in the study of MMMs. This analysis helps in understanding the chemical interactions and the distribution of functional groups within the MMMs. This technique is essential for confirming the incorporation of fillers into the polymer matrix and for examining the interfacial interactions between the polymer and the fillers.

For instance, in the development of PVDF based on MMMs, FTIR spectroscopy was used to confirm the successful integration of NPs like TiO_2_ and ZIF-8. The characteristic peaks in the FTIR spectra provide insights into the chemical bonding and compatibility among the polymer matrix and the embedded fillers. Changes in the intensity and position of specific peaks can indicate successful filler incorporation and potential interactions between the polymer chains and the filler material. FTIR characterization also aids in evaluating the structural and physicochemical properties of the MMMs. It helps in identifying the functional groups present and ensures that the desired chemical modifications have been achieved. This is crucial for tailoring the membrane properties for specific applications, such as improving permeability, selectivity, and physical and mechanical strengths [74,75,76]. 

In summary, FTIR spectroscopy is an indispensable tool in the characterization of MMMs, providing critical information on the chemical composition and interactions within the membranes, thereby aiding in the optimization and development of high-performance MMMs for various industrial applications. 

### 4.3. Performance Characterization

Performance characterization of MMM is essential to evaluating their effectiveness in specific applications, such as gas separation, water purification, and PV. Key performance metrics include permeability, selectivity, and stability. Gas permeation tests measure the membrane’s ability to separate different gases based on their permeabilities, providing insights into the efficiency and selectivity of the MMM. Additionally, water flux and rejection rate tests are crucial for assessing the performance of MMMs in water treatment applications. Long-term stability tests under operational conditions are also conducted to ensure that the membrane maintains its performance over time. These comprehensive evaluations help optimize the MMM’s design and formulation, ensuring they meet the desired application requirements effectively and reliably. MMMs are characterized by various performance metrics that assess their efficiency in separation processes. Key performance parameters include pure water flux, fouling, and permeation studies.

#### 4.3.1. Pure Water Flux (PWF)

PWF is a fundamental parameter that measures the membrane’s permeability to water under a given pressure. This metric is crucial as it indicates the membrane’s efficiency in allowing water to pass through while retaining other substances. For MMMs, an enhancement in pure water flux often signifies improved porosity and hydrophilicity due to the addition of fillers. For example, incorporating hydrophilic NPs into the polymer matrix can create additional pathways for water molecules, thereby increasing the flux. The characterization of PWF in MMMs is crucial for evaluating their performance, particularly in water treatment applications. PWF is typically measured by passing DI water through the membrane under controlled pressure and recording the volume of water that permeates per unit area of the membrane over time. This measurement provides insights into the membrane’s permeability and hydrophilicity, which are essential parameters for assessing its suitability for various filtration processes.

For instance, studies have shown that incorporating hydrophilic additives, or NPs, into the polymer matrix can significantly enhance the PWF of MMMs. This improvement is because of the improved hydrophilicity and the creation of more uniform and interconnected pore structures within the membrane. For example, in a study where TiO_2_-NPs were added to a PVDF membrane, the modified membrane exhibited a notably higher PWF than the pristine PVDF membrane, indicating better water permeability and fouling resistance [77]. 

Overall, PWF characterization is an essential step in developing and optimizing MMMs, as it directly influences their efficiency and applicability in water filtration and treatment processes. 

#### 4.3.2. Fouling Studies

Fouling characterization of MMM is crucial to evaluating their performance and longevity, particularly in filtration applications. Fouling typically manifests as a decline in membrane permeability due to particles, organic compounds, or biofilm accumulation on the surface of the membrane, which in turn leads to increased operational costs and reduced efficiency.

Several methods and metrics are employed to characterize fouling. One common approach is measuring the decline in flux over time during filtration. This involves recording the pure water flux before and after filtering a foulant solution, allowing for the calculation of the flux recovery ratio (FRR). A higher FRR indicates better fouling resistance. Additionally, the resistance-in-series model can quantify fouling resistance, such as cake layer resistance, pore blocking, and adsorption within the membrane pores.

Another crucial parameter is the critical flux, which denotes the flux level at which fouling becomes negligible. This is determined by incrementally increasing the flux until a rapid transmembrane pressure (TMP) increase is observed. Reports have shown that optimizing the functions of MMMs, such as surface hydrophilicity, and incorporating antifouling agents like TiO_2_ or GO can significantly improve their fouling resistance.

Advanced techniques like SEM and AFM give a detailed understanding of the fouling layer’s structure and thickness. At the same time, spectroscopic methods like ATR-FTIR can identify the chemical composition of foulants. By employing these characterization techniques, researchers can better understand fouling mechanisms and develop more effective MMMs for various filtration applications [78].

#### 4.3.3. Permeation Studies

Permeation studies focus on the membrane’s ability to separate specific gases or liquids from mixtures. These studies measure parameters such as selectivity and permeability for targeted components. For example, MMMs are tested for their ability to selectively permeate gases like CO_2_ or O_2_ over other gases such as N_2_ in gas separation applications. The presence of fillers like MOFs or carbon-based materials can significantly enhance the selective permeation properties of MMMs by providing additional or preferential pathways for certain molecules [75].

## 5. Applications of MMMs in Separation Processes

**Figure 8:**
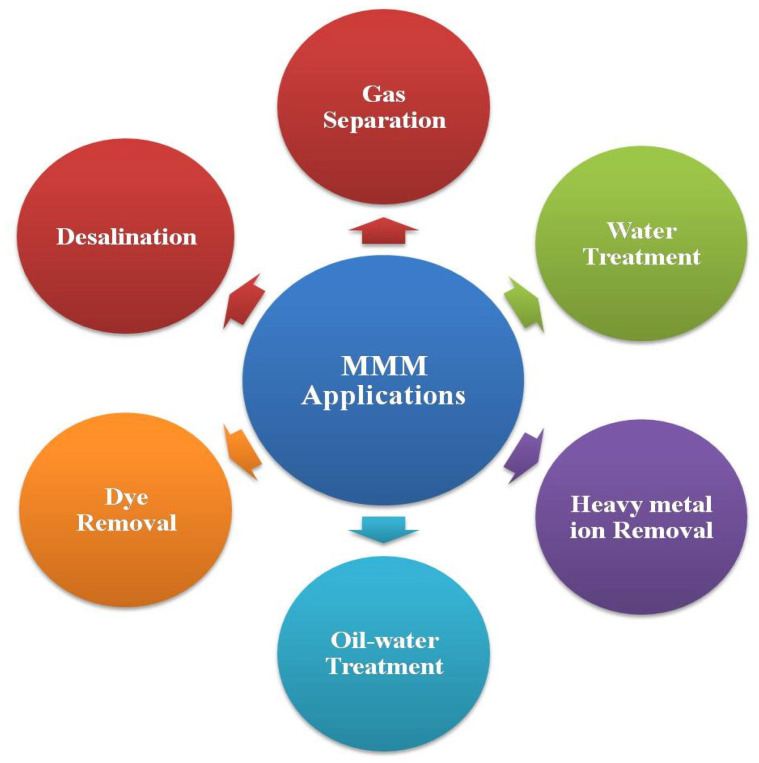
Various applications of MMM in separation processes.

MMMs are used extensively across various applications due to their improved separation capabilities, resulting from the combined effects of polymer matrices and inorganic fillers. In water treatment, MMMs significantly enhance the removal of contaminants like heavy metals, organic pollutants, and salts by boosting the membranes’ antifouling properties and mechanical robustness. For gas separation, MMMs increase the selectivity and permeability of gases such as CO_2_, CH_4_, and H_2_, making them indispensable in the natural gas processing and carbon capture industries. Additionally, MMMs play a critical role in PV, effectively separating azeotropic mixtures and organic compounds and aiding in solvent dehydration. These advancements render MMMs highly valuable for desalination, wastewater treatment, and potable water production [25]. Applications of MMM in various fields are shown in Figure 8.

### 5.1. Water and Wastewater Treatment

#### 5.1.1. Water Purification

Meeting the growing need for clean water owing to urbanization and industrial growth has made water and wastewater treatment a top priority in recent years [79]. Water treatment is vital for providing clean water for various purposes, including drinking, industry, agriculture, and recreation, ensuring the ecosystem’s health. Membrane processes offer significant advantages over traditional methods and are widely used in water purification. These processes, like MF, UF, NF, and RO, are tailored based on water composition and effluent type, making them integral to modern water treatment systems [80].

**Figure 9:**
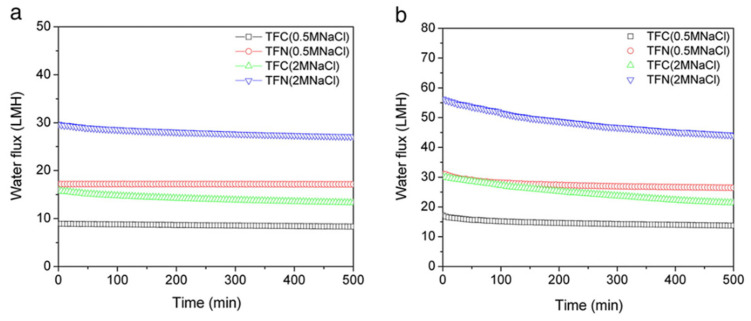
Water flux performance of TFC and TFN membranes over time with varying concentrations of the draw solution (**a**) AL–FS and (**b**) AL–DS orientations (test conditions: feed solution: 10 mM NaCl, cross-flow velocity: 350 mL/min on both sides of the FO membrane, and temperature: 25 °C). (Reproduced from Ref. [81] with permission).

In today’s water treatment scenario, membranes integrated with NPs serve as essential components in filtration systems, significantly improving contaminant removal from wastewater. By incorporating NPs, these membranes effectively mitigate issues such as fouling while enhancing overall process efficiency. For example, a study by Emadzadeh et al. demonstrated that including TiO_2_-NPs in PSF membranes enhanced water purification and maintained salt rejection levels, showcasing the potential of NP integration in membrane technology [81,82]. The water flux of thin-film composite (TFC) and thin-film nanocomposite (TFN) membranes is shown in Figure 9 as a function of time at different concentrations of the initial draw solution for both AL–DS and AL–FS orientations, where the active layer faces the draw solution and the feed solution, respectively. It is evident that both membranes’ water flow tended to decrease with longer filtering periods for the AL–DS orientation. The flux reduction was more significant, especially for the 2 M NaCl draw solution, where the TFN membrane’s flux decreased by 22% and the TFC membrane’s by 29% at the conclusion of the filtering time. Compared to standard TFC and commercial cellulose triacetate (CTA) membranes, the TFN membrane consistently exhibited significantly higher forward osmosis (FO) water flux without a substantial increase in reverse solute flux under identical conditions. In the AL–FS configuration, with 0.5 M NaCl as the draw solution and 10mM NaCl as the feed solution, the water flux of the TFN membrane exceeded that of the commercial membrane by roughly 120% and surpassed that of the typical TFC membrane by approximately 87%. Dong et al. pioneered the development of high-flux RO membranes by leveraging NaY zeolite NPs specifically tailored for desalinating brackish water sources [83]. Gahlot et al. thoroughly assessed a nanocomposite ion exchange membrane (IEM) consisting of sulfonated PES blended with different proportions of GO-NPs. Their investigation revealed a remarkable 300% enhancement in water permeability while maintaining comparable NaCl rejection rates. This improvement was attributed to the heightened membrane hydrophilicity achieved through the strategic integration of GO-NPs [82,84]. Table 1 summarizes key findings of NP-loaded MMMs in water treatment, detailing their flux, bacterial removal efficiency, and rejection performance.

#### 5.1.2. Antifouling and Antibacterial Activity

Membrane fouling poses a significant challenge due to direct contact with feed water, which contains numerous impurities. This fouling can result from various mechanisms, including molecular adsorption, particulate deposition on the membrane surface, and microbial adhesion. Consequently, the accumulation of feed components reduces the efficiency of mass transport [92]. This leads to a decrease in permeate flux, an increase in transmembrane pressure, a reduction in filtration efficiency, and ultimately a shorter membrane lifespan. Utilizing mixed-matrix membranes (MMMs) with nanomaterials presents a promising approach to enhancing the performance of various membrane processes [9]. Bassyouni et al. published a comprehensive review of the use of nanocomposite membranes and their effects on the overall performance of membrane processes. Researchers have investigated the incorporation of various nanomaterials as additives within polymers to enhance hydrophilicity, reduce membrane fouling, and improve membrane performance [93]. 

Incorporating TiO_2_ NPs into the dope solution offers numerous benefits, such as enhanced hydrophilicity, reduced fouling, and improved thermal and mechanical stability of composite membranes. By adjusting the concentrations of TiO_2_-NPs within different polymer matrices, like polyphenylsulfone (PPSU), these advantages can be optimized. Beyond their antifouling capabilities, TiO_2_ nanoparticles are appreciated in membrane technology for their accessibility, antibacterial properties, cost-effectiveness, and exceptional stability [94,95,96]. 

Bae et al. provided experimental evidence showcasing reduced membrane fouling by incorporating TiO_2_-NPs in NF membranes, utilizing a membrane bioreactor (MBR) system. Meanwhile, Vetrivel et al. demonstrated the efficacy of hydrous manganese dioxide (MnO_2_) NPs embedded in cellulose acetate (CA) membranes. Their study highlighted notable outcomes, including a high flux recovery rate, irreversible fouling mitigation, permeate water flux maintenance, and effective rejection of bovine serum albumin (BSA) [97,98]. 

Behboudi et al. significantly improved the antifouling characteristics of polyvinyl chloride (PVC)/polycarbonate (PC)/modified Ag-NPs HFMs using the Stober method, specifically for treating pharmaceutical wastewater. Their approach led to an impressive 98.1% removal of chemical oxygen demand (COD). Meanwhile, Zodrow et al. synthesized UF membranes by blending PSF and PVP with Ag-NPs. This resulted in enhanced flux rates and increased antibacterial properties compared to untreated PSF membranes [99,100]. Graphene-based nanocomposites have garnered interest for their antimicrobial properties and potential in drug delivery applications. However, despite these promising traits, graphene-related materials in biomedicine encounter various challenges that must be addressed for future research in the field [101,102]. 

#### 5.1.3. Dye Removal

**Figure 10:**
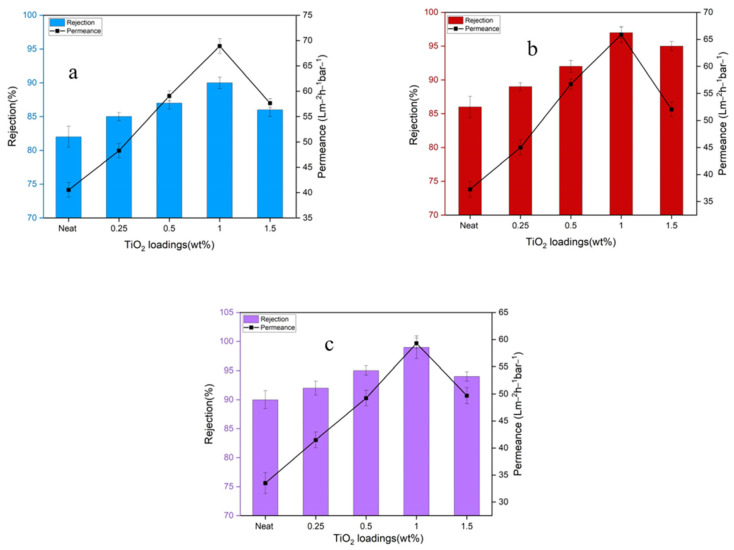
(**a**) MB rejection, (**b**) CR rejection, and (**c**) RB rejection [107].

Dyes represent the most prevalent organic pollutants in industrial effluents, posing significant toxicity risks to both plant and animal life. Their effective removal is crucial for ecosystem sustainability, yet they often resist degradation through photolysis and conventional wastewater treatment methods [103]. Textile dyes are characterized by their intricate aromatic structure, which features delocalized electrons and conjugated double bonds. These properties contribute to their chemical stability and make them resistant to degradation in the natural environment, posing persistent challenges or remediation efforts [104]. Membrane technology plays a crucial role in dye filtration and separation. Inorganic and MMMs, known for their high porosity, enhanced stability, improved permeability, superior selectivity, and resilience to harsh chemical and thermal conditions, have shown particular promise for dye removal compared to polymeric membranes [103]. The textile industry, known for its extensive use of water and diverse chemicals, is a significant source of water pollution globally. In addressing this challenge, Zheng et al. introduced a novel approach by creating hollow fiber NF membranes. Their innovation involved combining polyquaternium-10 and glutaraldehyde with polyvinyl alcohol (PVA) and polypropylene (PP). These membranes exhibited remarkable efficiency in removing various dyes commonly found in textile wastewater, such as Brilliant Green (BG), Victoria Blue B (VB), and Crystal Violet (CV), achieving impressive removal rates of 99.8%, 99.8%, and 99.2%, respectively [82,105]. Table 2 details the performance of various NPs incorporating MMMs in dye removal. Kadhim et al. developed mixed-matrix membranes (MMMs) by modifying polyether sulfone (PES) with graphene oxide (GO) to remove toxic dyes from Rose Bengal and Acid Black. They found that increasing GO content enhanced the membrane’s permeate flux, with 0.5 wt% GO yielding the lowest contact angle, highest porosity, and average pore size, resulting in a maximum water flux of 116.5 L/m^2^ h. However, further increasing GO content reduced water flux due to pore blockage, with porosity dropping from 80.6% at 0.5% GO to 65.4% at 1.5% GO. The membranes achieved a rejection rate above 99% for both dyes, attributed to the repulsive force from negative charges on the membrane surface due to GO’s functional groups. Additionally, the membrane with 0.5 wt% GO showed improved long-term performance, sustaining a higher flux over 26 days compared to 14 days for the control membrane [106]. Ayesha et al. incorporated titanium dioxide nanotubes (TiO_2_NTs) into PES nanofiltration membranes to enhance hydrophilicity. The resulting MMMs were tested for water permeability and solute rejection. MMMs with varying TiO_2_NT loadings were evaluated for dye removal efficiency using Methylene Blue (MB), Congo Red (CR), and Rose Bengal (ROB). As represented in Figure 10 membranes, with 1 wt% TiO_2_NT showed high rejection rates of 82–90% for MB, 86–97% for CR, and 90–99% for ROB, with rejection increasing with dye molecular weight. The incorporation of TiO_2_NTs improved solute rejection and water flux, highlighting their effectiveness in blocking dye molecules. Additionally, the interactions between the membrane surface and dyes influenced dye rejection, with positively charged MB attracted and negatively charged CR and ROB repelled [107]. 

#### 5.1.4. Heavy Metal Removal

Water contamination by harmful metal ions poses a significant environmental challenge, affecting human health and ecosystem integrity globally. With rising populations and accelerating industrialization, the discharge of heavy metals into water bodies has increased due to agricultural practices, mining, semiconductor manufacturing, electroplating, and paint industries. Heavy metals, known for their extreme toxicity and classification as “Class A” human carcinogens, make drinking water contamination a severe threat to global water security, causing serious health issues. Conventional membrane filtration for heavy metal removal is energy-intensive and often requires post-treatment mineral adjustments. This has led to attempts to ban reverse osmosis in some regions. Therefore, developing a new generation of membrane materials for water filtration is essential to address these challenges [120]. 

**Figure 11:**
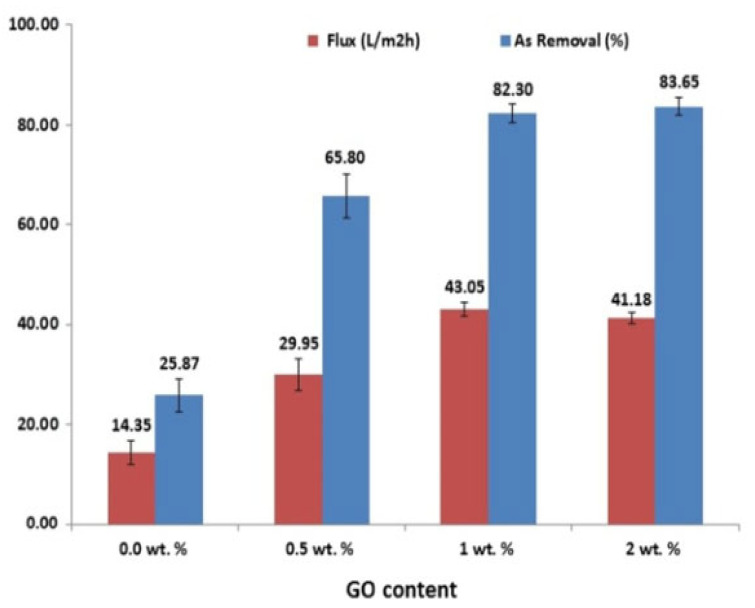
Rejection of As(V) and flux of the prepared membranes with different GO concentrations. (Reproduced with permission from Ref. [72]).

While traditional approaches such as ion exchange and filtration have historically been employed to eliminate toxic heavy metals from water, contemporary membrane technology is emerging as a more environmentally sustainable alternative. Despite facing energy consumption hurdles, surface-modified membranes offer a hopeful avenue. By integrating nanomaterials, these membranes undergo enhancements in mechanical robustness and metal rejection capabilities. Notably, multivalent metals like chromium (Cr), arsenic (As), and selenium (Se) are efficiently extracted using positively charged adsorbents, whereas divalent ions are attracted to their negatively charged counterparts. The surface charge of the adsorbent plays a pivotal role in governing the efficacy of adsorption processes [121]. In their study, Rezaee and colleagues fabricated PSF-NCMs by incorporating GO-NPs using the PI method to separate arsenate pollutants from water. The fabricated membranes based on GO content are named pure PSF (0.0 wt%), PSF/GO-0.5 (0.5 wt%), PSF/GO-1 (1 wt%), and PSF/GO-2 (2 wt%). Their findings highlighted that incorporating 1 wt% of NPs notably enhanced hydrophilicity [72,82]. Figure 11 illustrates the outcomes of arsenate rejection and membrane flux for various membranes. The pure water flux for PSF/GO-1 was determined to be approximately 50 L/m^2^ h at 4 bar. Meanwhile, the PSF/GO-2 membrane exhibited the highest rejection rate, reaching approximately 83.65% at 4 bar. Additionally, it was found that arsenate rejection varied with the solution’s pH, with higher pH levels improving rejection rates. This study demonstrated that incorporating GO into the PSF casting solution could enhance membrane hydrophilicity, porosity, flux, and arsenate rejection [72].

Jeon J et al. developed a specialized membrane known as Chi@Fe_2_O_3_-PVDF, comprising chitosan-coated iron oxide NPs immobilized on a hydrophilic PVDF matrix. This membrane showcased remarkable adsorption capabilities, particularly for Cr (VI), highlighting its potential for efficiently removing chromium ions from aqueous solutions [122]. 

### 5.2. Gas Separation

**Figure 12:**
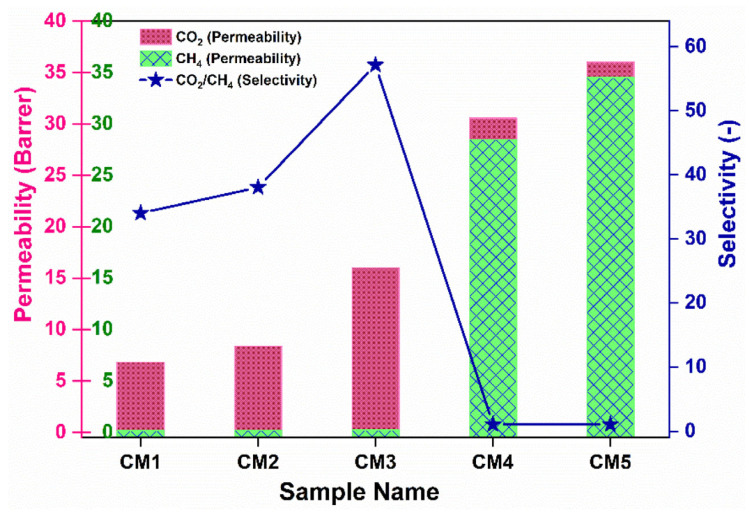
Single gas separation performance of synthesized mixed-matrix membranes at different concentrations of MXene nanofillers. Adopted from [127].

Gas separation processes commonly utilize dense membranes to filter gas molecules selectively based on size, diffusivity, or solubility. The choice of an appropriate membrane depends on the desired purity level of the separated gases. Membrane separation technology is widely used for gas purification but faces challenges such as high material costs and limitations in permeability and selectivity. Developing high-performance membrane materials with excellent stability and mechanical strength is essential. Due to their energy efficiency and low cost, polymer membranes are important for gas separation but are limited by the “trade-off” effect. Inorganic membranes offer superior separation performance and stability and are costly and difficult to manufacture without defects [123]. To address this issue, a primary strategy currently involves incorporating inorganic fillers into polymers to create MMMs. MMMs represent an innovative membrane material that combines a polymer matrix as the continuous phase with inorganic particles as the dispersed phase [124]. This approach combines the distinct benefits of inorganic particles for gas adsorption and separation with the excellent processing performance of polymer materials. Xiaoyu Tan et al. utilized a modified evaporation-induced phase-separation technique to develop an MMM to enhance CO_2_ separation efficiency. The MMM incorporated Na-SSZ-39 zeolite into a commercial polyimide, achieving ultrahigh (>50 wt%) zeolite loadings. This innovative approach created a percolating gas permeation highway within the membrane, significantly improving CO_2_ removal performance. The resulting MMM showed superior performance to polymeric and zeolite-only membranes, demonstrating robustness and scalability for economical deployment in various gas and liquid separations, particularly with challenging zeolites [23]. Membrane performance is typically evaluated regarding permeance and selectivity, often measured in the Barrer and Gas Permeance Unit (GPU). Membrane selectivity represents the ratio of gas permeability through a selective membrane in a binary mixture, providing a crucial metric for assessing separation efficiency [125]. In the 1980s, PDMS membranes were initially employed for gas separation, primarily due to their economic advantages. However, in recent times, membranes incorporating NPs have emerged as a preferred choice for CO_2_ separation in various industries, including the natural gas, refinery, and petrochemical sectors. This shift in preference towards nanoparticle-enhanced membranes is attributed to their superior performance and efficiency in CO_2_ separation processes [126]. Regmi et al. incorporated two-dimensional, multi-layered Ti_3_C_2_T_x_ MXene nanofillers to enhance the compatibility and CO_2_/CH_4_ separation performance of cellulose triacetate (CTA)-based mixed-matrix membranes (MMMs). As illustrated in Figure 12, the pure gas permeance for both CO_2_ and CH_4_ gradually increased with the addition of MXene up to 5 wt%, with both gases showing improved permeance. While CH_4_ permeance significantly increased at higher MXene loadings, a notable rise was observed up to 3 wt%. Consequently, the CO_2_/CH_4_ selectivity peaked at 3 wt% (reaching 57.14) before decreasing to 5 wt%. Compared to the CO_2_ permeance of pristine CTA (3.01 Barrer), the CTA sample with 3 wt% MXene showed a 5-fold increase in permeability, reaching 16 Barrer, along with a 2-fold increase in CO_2_/CH_4_ selectivity. Thus, introducing MXene nanosheets up to 3 wt% provides additional molecular transport channels, enhancing both CO_2_ permeance and CO_2_/CH_4_ selectivity [127]. 

In their research, Jusoh et al. developed a mixed-matrix membrane by incorporating Zeolite T into 6FDA-durene polyimide for CO_2_ separation. This study found a significant CO_2_/CH_4_ separation enhancement with the fabricated MMM. Specifically, incorporating just 1 wt% of Zeolite T led to an 80% increase in permeability [128,129]. In their experiments, Tavasloli et al. demonstrated that PSF-MMMs loaded with hexamethylenetetramine dicyanamide cadmium-MOF (2.5 wt%) exhibited exceptional selectivity for CO_2_/CH_4_, CO_2_/N_2_, and O_2_/N_2_ compared to pure PSF membranes [130]. Meanwhile, Gobi et al. achieved a remarkable 48% oxygen purity utilizing SiO_2_-NP-incorporated PAN, CA, and PEG membranes produced through the electrospinning technique [131]. Additionally, Farrokhnia et al. synthesized α alumina-filled PES-NCMs for H_2_, N_2_, and CO_2_ gas separation, revealing that H_2_ permeability and selectivity were amplified by eight times and 2.4 times, respectively, compared to pristine PES membranes [132]. Table 3 presents the performance analysis of NP-loaded MMMs for gas separation applications.

### 5.3. Membrane Distillation (MD) and Forward Osmosis (FO)

MD and FO present two unique methods for separation processes, each offering distinct advantages. MD employs a porous hydrophobic membrane, capitalizing on differences in vapor pressure. It encompasses various types, such as contact area, air gap, sweeping gas, and vacuum MD. Membrane distillation (MD) is notable for its minimal energy utilization and minimal investment requirements, making it appropriate for solvent dehydration, desalination, and chemical recovery [137,138]. Polymeric materials like PTFE, PVDF, PP, and PE are commonly used in membrane distillation (MD) due to their hydrophobicity, cost-effectiveness, and ease of fabrication. However, they face challenges such as limited thermal and chemical stability, which can lead to degradation in high-temperature or harsh chemical environments. Ceramic membranes, made from materials like alumina, zirconia, and titania, offer superior stability and resistance but are more expensive and prone to mechanical damage. MD technology has advanced significantly, offering an energy-efficient and cost-effective solution for seawater desalination and wastewater treatment. Recently, nanotechnology has transformed MD membranes, introducing nano-enabled membranes that combine the benefits of polymers and ceramics. Mixed-matrix and nanocomposite membranes, which integrate inorganic fillers into polymers, enhance stability while maintaining cost-effectiveness and ease of processing [139]. FO leverages differences in solute concentration for separation, making it valuable in desalination, power production, and numerous industrial applications. Forward osmosis (FO) has recently gained attention in desalination for its ability to separate pure water from salt, sea, and brackish water using natural osmotic pressure without external energy, unlike reverse osmosis (RO). FO membranes are advantageous due to their low cost and energy efficiency, making them suitable for landfill leachate treatment, emergency water supplies, and seawater desalination. High-performance FO membranes are often made from interfacial polymerization, which provides excellent permeability and pH tolerance. Despite their benefits, traditional thin-film composite (TFC) membranes used in FO face challenges like limited water flux, reverse solute flux, and fouling. Recent advancements have focused on modifying TFC membranes with various nanomaterials to address these issues, leading to the development of thin-film nanocomposite (TFN) membranes [140]. The advent of NCMs has transformed FO, especially in tackling issues such as bio-fouling and internal concentration polarization (ICP). These advancements have been particularly significant in industries like fruit juice concentration and beverage production, where maintaining high-quality filtration is crucial [141,142].

NP integration into membranes represents a significant advancement in both MD and FO processes. Golamzadeh et al. developed a PES membrane infused with oleic acid-treated cobalt oxide (Co_3_O_4_)-NPs, achieving outstanding salt rejection (>99.8%) and a high permeation flux of 11.6 L/m^2^ h in MD [143]. Meanwhile, Cheng et al. showcased the enhanced performance of hydrophobic fluorinated membranes by incorporating aluminum fumarate MOF into PVDF polymers. This integration resulted in salt rejection permeability exceeding 99.9% and 50.5%, attributed to improvements in mass transfer coefficient, thermal efficiency, and membrane porosity [144]. Silva et al. explored direct contact MD, enhancing flux to 9.5 × 10^−3^ kg/m^2^ h and 100% salt rejection by incorporating MWCNT and PVP into PVDF membranes [145]. 

In the realm of FO, Wang et al. conducted studies incorporating TiO_2_ and Al_2_O_3_ nanofillers into PSf membranes, resulting in remarkable flux enhancements of 8.25 L/m^2^ h and 27.6 L/m^2^ h, respectively, alongside low solute reverse flux. This research underscores the transformative potential of nanoparticle (NP)-modified membranes in FO applications [146]. Similarly, Liu et al. achieved enhanced flux (9.31 L/m^2^ h) by introducing calcium carbonate (CaCO_3_) NPs into thin-film composite (TFC) membranes, demonstrating the efficacy of NP integration in improving membrane performance [147]. Moreover, Darabi et al. synthesized a TF-NC membrane with Fe_2_O_3_ incorporation, elevating flux from 10.4 to 17.5 L/m^2^ h while mitigating internal concentration polarization (ICP). These findings highlight the substantial advancements and potential of NP-modified membranes across diverse industries, promising innovative solutions for separation processes in MD and FO [148]. 

### 5.4. Pervaporation (PV)

PV is a separation method that utilizes vacuum pressure to drive the “solution-diffusion” process through a non-porous membrane, efficiently separating volatile substances by exploiting differences in partial pressures. This method proves particularly useful for separating azeotropic mixtures, heat-sensitive compounds, and volatile organic compounds (VOCs) in an eco-friendly manner. NCMs (NCMs) have garnered interest in PV applications, aiming to enhance membrane properties and improve separation efficiency. This focus often centers on leveraging nanoscale pore networks within the diffusion-sorption mechanism. PV presents a cost-effective alternative to energy-intensive methods like azeotropic distillation, making it a promising solution for various separation processes [149,150]. NCMs exhibit a wide array of applications in permeation vaporization, from separating organic–organic mixtures to dehydrating organic solvents and recovering organics from aqueous solutions and alcohols. Metal-organic framework (MOF)-based mixed-matrix membranes (MMMs) have gained significant attention for pervaporation due to their enhanced selectivity, increased permeability, and improved mechanical strength. MMMs, which consist of a polymer continuous phase and an inorganic filler discrete phase, leverage the advantages of both materials. MOFs, first conceptualized in 1995, are particularly valued for their high surface area, recyclability, and permanent porosity. Within MMMs, MOF particles act as molecular sieves, enhancing selectivity and improving permeation by modifying polymer chain packing and increasing free volume. This results in MMMs that outperform traditional polymer membranes by achieving superior separation performance in permeability and selectivity [151]. Sudhakar et al. achieved notable advancements in PV efficiency by integrating 13X Zeolite fillers into chitosan polymer matrices. Their optimized membrane, comprising 30 wt% 13X zeolite and 2 wt% chitosan, showcased superior selectivity (1620), marking a significant enhancement in performance [152]. Similarly, Majid Pakizeh et al. synthesized GO/PVA MMMs with different GO loadings to separate toluene/n-heptane. The membrane containing 1.5 wt% GO performed best, achieving a total permeation flux of 30.6 g/m^2^ h and a separation factor of 11.9. Additionally, this membrane had a pervaporation separation index of 333.54, representing a 199% improvement over the pure PVA membrane, which had an index of 111.4 [153]. Table 4 provides a detailed comparison of polymeric membranes loaded with various NPs for pervaporation applications.

## 6. Challenges and Future Directions

The advancement of performance enhancement strategies in MMMs faces several key challenges and future directions. First, ensuring consistent dispersion of fillers within the polymer matrix remains a significant challenge, particularly for advanced materials like MOFs and PAFs. Overcoming this hurdle requires innovative mixing techniques and surface modification strategies to enhance compatibility between fillers and matrices. Additionally, ensuring strong interfacial bonding between the polymer and fillers is crucial for maximizing membrane performance and durability. Future research should focus on developing novel surface chemistries and functionalization methods to optimize these interactions. Moreover, while current fabrication techniques offer precise control over membrane properties, scalability, and cost-effectiveness remain limiting factors for industrial adoption. Streamlining manufacturing processes and exploring new production methods, such as continuous fabrication techniques, could address these challenges and facilitate the widespread implementation of MMMs in various applications. Lastly, ongoing research should prioritize sustainability and environmental impact, emphasizing the development of eco-friendly materials and fabrication processes to minimize resource consumption and waste generation, ultimately contributing to a more sustainable future for membrane technology.

Challenges and future directions in applying MMMs in separation processes encompass several key areas. Addressing fouling and bacterial contamination remains a significant challenge, necessitating further research into innovative antifouling strategies and antibacterial properties. Dealing with complex textile dyes and efficiently removing heavy metals from water requires advanced membrane materials and surface modifications to achieve higher selectivity and permeability. Improving the selectivity and permeability of membranes for specific gases like CO_2_, CH_4_, and H_2_ is crucial in gas separation, necessitating the development of novel MMM with enhanced performance. Optimizing PEMFCs through nanomaterial integration for improved conductivity and selectivity presents a promising avenue for enhancing fuel cell technology. Additionally, further advancements in NCMs are needed to overcome challenges such as biofouling and internal concentration polarization (ICP) for more efficient separation processes in MD and FO techniques. Research focus in PV applications should be directed towards enhancing the selectivity and permeability of NCMs for separating volatile compounds, offering a cost-effective alternative for various separation processes. Addressing these challenges and exploring future directions will contribute to the continued advancement and adoption of MMMs in separation processes, catering to the increasing demand for clean water, efficient gas separation, and sustainable energy production.

## 7. Conclusions

In conclusion, integrating membrane technology and nanomaterials holds significant promise for advancing selective transport processes and industrial separations. Membranes act as crucial barriers, regulating the transport of components between phases and serving diverse functions across various applications, such as wastewater management and desalination. Despite their benefits, challenges such as fouling and concentration polarization persist, which can be mitigated by innovations like NCMs. MMMs, which combine polymer matrices with inorganic or organic fillers, exemplify a cutting-edge development in this field, enhancing permeability, selectivity, and durability. Advanced synthesis techniques, like phase inversion, electrospinning, and layer-by-layer assembly, have been instrumental in optimizing membrane properties. These methods allow for precise control over filler dispersion and interfacial bonding, which is crucial for achieving high-performance membranes. Moreover, characterizing MMMs through techniques like SEM, TEM, XRD, and FTIR provides insights into their structural integrity and separation efficiency, guiding further improvements. While challenges such as filler aggregation and scalability remain, ongoing research and innovation continue to address these issues. As the field progresses, the synergy between membrane technology and nanomaterials is expected to drive significant advancements in sustainable and efficient separation processes, meeting the growing demands of various industries.

## Figures and Tables

**Table 1:** Key findings of NP-loaded MMMs in water treatment.

S. No	Nanoparticles	Polymer	Key Findings	References
1	Ag-NPs	PES + PVP	Enhanced hydrophilicity, water permeability, protein flux, and BSA rejection	[85]
2	Cu-NPs	PES %	Superior antifouling and protein rejection compared to a pristine PES membrane	[86]
3	CuO-NPs	TFC-RO membrane on PSf substrate	Improved anti-biofouling but compromised desalination performance	[87]
4	Alumina (Al_2_O_3_) and Linda type L (LTL) zeolite NPs	PSF and PVP	Greater anti-adhesion efficiency to either *P. aeruginosa* or *E. coli*	[88]
5	Iron oxide (FeO)	PES	Enhanced hydrophilicity, flux, and salt rejection.	[89]
6	Zeoliticimidazolate framework-8 (ZIF-8)	PVDF	Increased water flux, high permeability, and FRR %	[90]
7	TiO_2_	PES	Enhanced PWF, antifouling, and low flux decline.	[91]

**Table 2:** NP-loaded MMMs for dye removal.

S. No	Type of Dye	Nanoparticles	Polymeric	% Removal	Ref
1.	Congo Red (CR)	Cobalt	PU	60	[108]
2.		ZnO	PSf/PVA	53.5	[109]
3.		Mesoporous silica	PVDF/PTFE	99%	[110]
4.		Hydrogen titanate	Chitosan	98.7	[111]
5.		TiO_2_	PVDF-co-hexafluoropropylene (HFP)	>99.8	[112]
6.	Indigo Dye	TiO_2_	PVDF	6.1 μmol L^−1^ min^−1^	[113]
7.		FeO-NPs	Sulfonated waste-expanded polystyrene	99%	[114]
8.	Methyl Orange (MO)	Hyperbranched polyethyleneimine (HPEI)-templated TiO_2_	PES	70.3%	[115]
9.	MO	ZnO	CA	75%	[116]
10.	MO, Methylene Blue (MB), and Rhodamine B (RhB)	Polydopamine (PDA)-immobilized TiO_2_	Bacterial cellulose (BC)	MO-95.1% MB-99.5% RhB-100%	[117]
11.	MO and Allura Red	Polyaniline-titanium nanotubes	PVDF	MO-90% AR-88%	[118]
12.	RhB	Copper sulfide (Cus)/gelatin	PVA	81%	[119]

**Table 3:** NP-loaded MMMs for gas separation applications: performance analysis.

S. No	Nanoparticles	Polymer	Selectivity	Reference
1	MWCNTs	PES + PEG + Pebax-1657	The selectivity of the modified membrane for the CO_2_/N_2_ gas pair improved from 83.2 to 162 as the feed pressure increased from 1 to 3 MPa. For the H_2_/N_2_ and O_2_/N_2_ gas pairs, the selectivity values ranged from 82.5 to 90 and from 7.1 to 6.8, respectively.	[133]
2	Zeolite 4A	Pebax-1657 + PES	The permeability increased from 71.4 to 155.7 Barrers for CO_2_, 2.2 to 19.6 Barrers for CH_4_, 5.8 to 17.9 Barrers O_2_, and 1.4 to 12.0 Barrers for N_2_. The selectivity improved from 54.1 to 94.2 for the CO_2_/N_2_, 26.4 to 41.3 for CO_2_/CH_4_, and 4.3 to 4.9 for the O_2_/N_2_ gas pair with increasing feed pressure from 5 to 25 kg/cm^2^.	[134]
3	Nanosilica and H-Mordenite	PES + Pebax-1657	The pressure increase from 10 to 30 kg/cm^2^ enhanced the permeance of the 0.3 wt% Si/Pebax membrane: CO_2_ (8.9 to 36.1 GPU), H_2_ (1.1 to 3.5 GPU), O2 (0.49 to 1.3 GPU), and N_2_ (0.12 to 0.43 GPU). The addition of H-Mordenite further increased permeance: CO_2_ (1.1 to 5.4 GPU), H_2_ (0.56 to 0.87 GPU), O_2_ (0.09 to 0.39 GPU), and N_2_ (0.03 to 0.205 GPU).	[135]
4	ZIF-8	Matrimid 5218	Enhanced gas permeability.	[136]

**Table 4:** Performance evaluation of NP-loaded MMMs for pervaporation.

S. No	NP	Polymer	Recovery of	Pristine Membrane Flux	Modified Membrane Flux	Separation Factor Improvement	Reason for Enhancement	Reference
1	ZIF-7	PDMS	Butanol from an aqueous solution	1080 g/m^2^/h	1689 g/m^2^/h	51–66	Enhanced the available space within the membrane matrix	[154]
2	TiO_2_ and TiO_2_ + Poly aniline (PANI)	PVA	Dehydration of IPA	PVA-TiO_2_ = 0.0221 kg/m^2^ h	PVA-TiO_2_-PANI = 0.0250 kg/m^2^ h	Selectivity is infinite for both membranes.	The presence of the emeraldine salt form of PANi increases hydrophilicity more than TiO_2_.	[155]
3	Dopamine-Ag (DAAg)	PDMS	Desulfurization	-	Three times the pristine PDMS membrane, i.e., 8.22 kg/m^2^/h	The enrichment factor is 5.03. It is 50% more than that of the PDMS membrane.	Facilitated transport of thiophene via reversible interaction with Ag(I) molecules increased separation performance by adjusting fractional free volume through DAAg NPs.	[156]
4	Nanosized silica particles with sulfonic acid groups (ST-GPE-S)	Chitosan	Dehydration of an ethanol–water solution	420 g/(m^2^ h)	410 g/(m^2^ h)	919	Silica nanoparticles increase water permeation in the chitosan polymer matrix by providing additional free volumes.	[157]
5	Ag-NPs	PVA	Dehydration of IPA	3.18 × 10^−2^ kg/m^2^ h	7.16 × 10^−2^ kg/m^2^ h	The separation factor improved from 244 to 634	Incorporating Ag NPs into the membrane enhances interactions between the NPs and the membrane.	[158]
6	Alumina NPs (Al_2_O_3_)	Cellulose triacetate (CTA)	Desalination of hypersaline solutions	2.2 kg/m^2^ h	6.7 kg/m^2^ h	Salt rejection is 99.8%	The required activation energy for water molecules increased from 34.1 to 43.2 kJ/mol.	[159]
7	Cerium oxide (CeO_2_)	PVA	Dehydration of ethanol	0.143 kg/m^2^ h	0.567 kg/m^2^ h	Increased from 51.2 to 1821	The presence of CeO_2_ NP in the PVA membrane increased the free water channels.	[160]

## Data Availability

No new data were created or analyzed in this study. Data sharing is not applicable to this article.
